# Publisher Correction: Future precipitation increase constrained by climatological pattern of cloud effect

**DOI:** 10.1038/s41467-024-48038-1

**Published:** 2024-04-29

**Authors:** Wenyu Zhou, L. Ruby Leung, Nicholas Siler, Jian Lu

**Affiliations:** 1 Atmospheric, Climate, and Earth Sciences Division, Pacific Northwest National Laboratory, Richland, WA USA; 2 College of Earth, Ocean, and Atmospheric Sciences, Oregon State University, Corvallis, OR USA

**Keywords:** Projection and prediction, Atmospheric dynamics, Hydrology

Correction to: *Nature Communications* 10.1038/s41467-023-42181-x, published online 11 October 2023

The copyright holder for this article was incorrectly given as ‘The Author(s)’ but should have been ‘Battelle Memorial Institute and Siler’.

