# Supplementary material for: Future precipitation increase constrained by climatological pattern of cloud effect
Source: Nat Commun. 2023 Oct 11;14:6363. doi: 10.1038/s41467-023-42181-x (PMC10567723; doi:10.1038/s41467-023-42181-x)
Supplement: Supplementary file 1 — Supplementary Information [file 41467_2023_42181_MOESM1_ESM.pdf]

Supplementary Information for

“Future precipitation increase constrained by climatological pattern of cloud effect”

Wenyu Zhou[1,*], Ruby Leung[1], Nicholas Siler[2], Jian Lu[1]

1. Pacific Northwest National Laboratory

2. Oregon State University

*wenyu.zhou@pnnl.gov

Table S1; Figures S1-S15

	Models
**CMIP5**	ACCESS1-0
	ACCESS1-3
	bcc-csm1-1
	BNU-ESM
	CanESM2
	CESM1-CAM5
	CNRM-CM5
	GFDL-CM3
	GISS-E2-H
	GISS-E2-R
	HadGEM2-ES
	IPSL-CM5A-LR
	IPSL-CM5B-LR
	inmcm4
	MIROC5
**CMIP6**	ACCESS-CM2
	ACCESS-ESM1-5
	BCC-CSM2-MR
	CESM2
	CMCC-CM2-SR5
	CNRM-CM6
	CanESM5
	EC-Earth3
	FGOALS-g3
	GFDL-CM4
	GISS-E2-1-G
	GISS-E2-1-H
	HadGEM3-GC31-LL
	IITM-ESM
	INM-CM4-8
	INM-CM5
	IPSL-CM6A-LR
	KACE-1-0-G
	MIROC6
	MRI-ESM2
	NorESM2-MM

Table S1: Models used in this study.

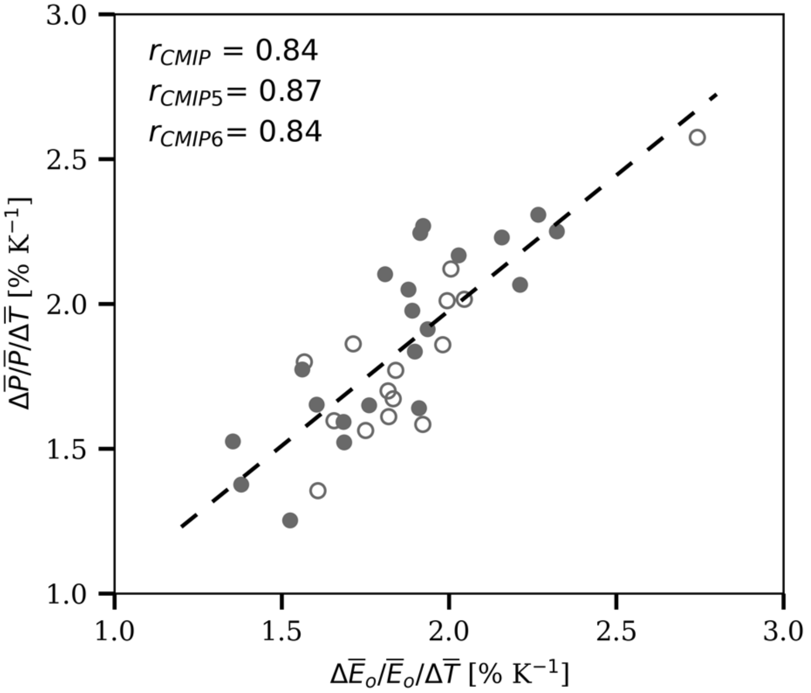

Figure S1: Intermodel scatterplot between the increase in global mean ocean evaporation per unit warming ($\Delta\bar{E}_o/\bar{E}_o/\Delta\bar{T}$) and the increase in global mean precipitation per unit warming ($\Delta\bar{P}/\bar{P}/\Delta\bar{T}$). The open and filled dots are the CMIP5 and CMIP6 results, respectively. The results are based on RCP8.5/SSP5-8.5 and future changes are computed as the change in climatology from the historical (1979-2005) to the future (2080-2100) period.

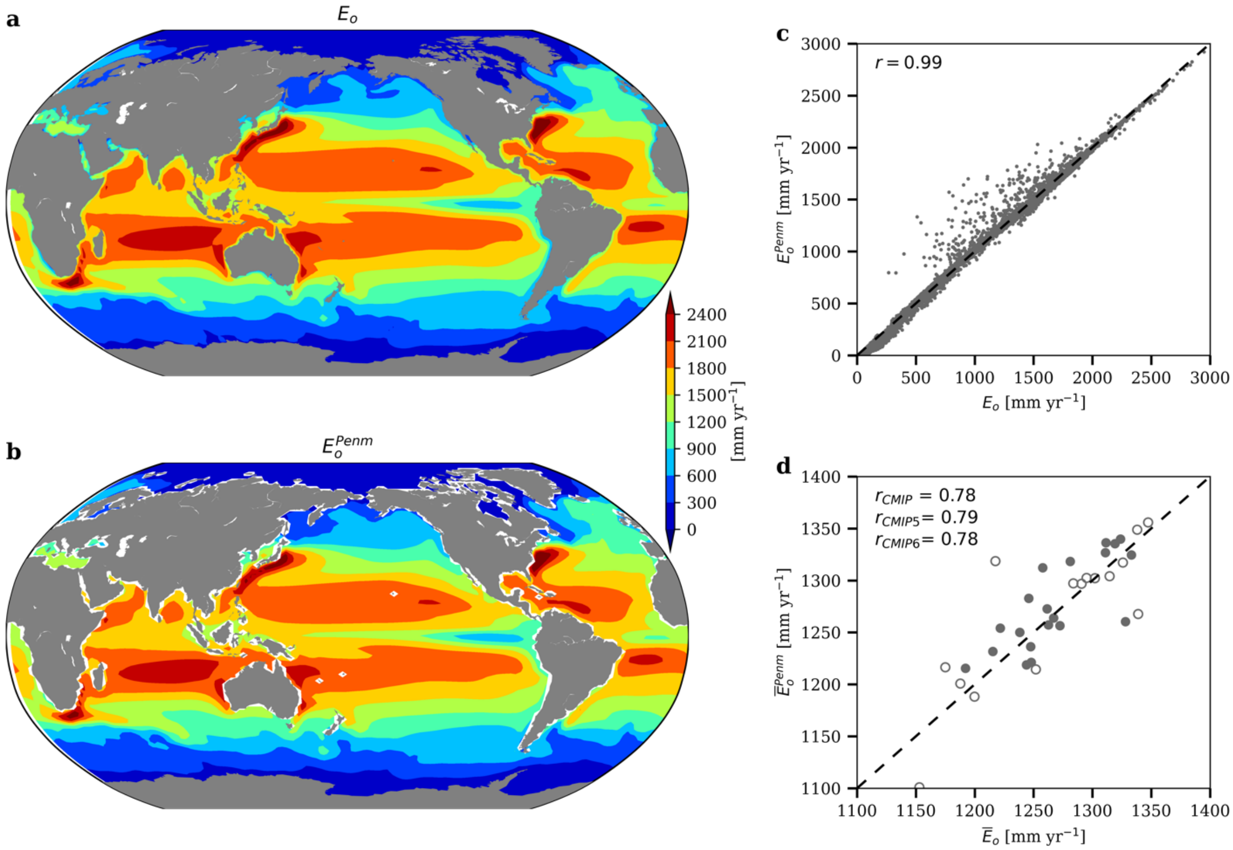

Figure S2: a, Spatial pattern of the simulated annual mean ocean evaporation in CMIP models (E_o). **b,** Spatial pattern of the estimated annual mean ocean evaporation using the Penman equation (E_o^{Penm}). **c,** Scatterplot between the simulated and estimated ocean evaporation. Each point corresponds to the value over a grid point. **d.** Intermodel scatterplot between the estimated and simulated global mean ocean evaporation. The open and filled dots are the CMIP5 and CMIP6 results, respectively.

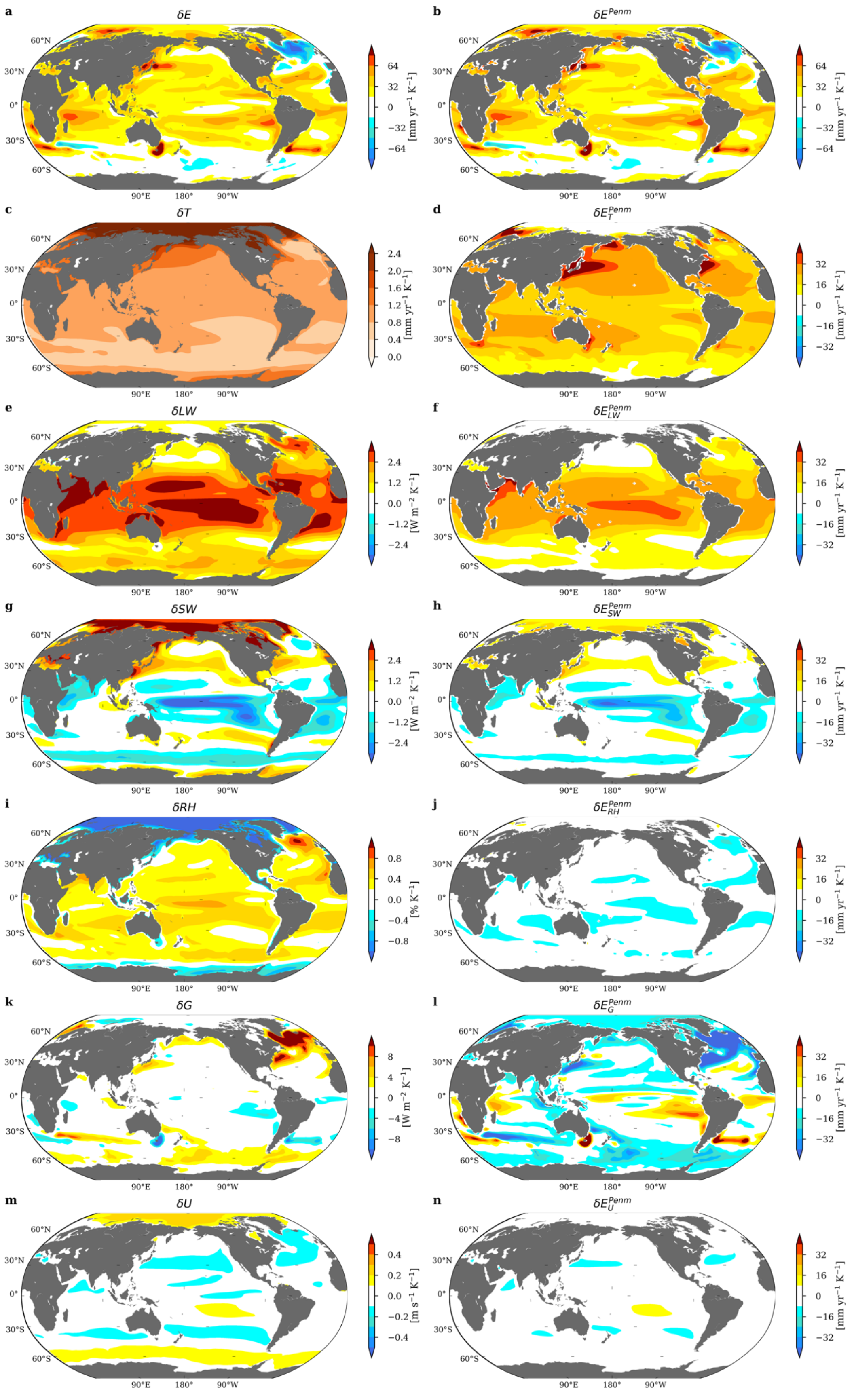

a
δE
b
δE^{Penm}
64
32
0
−32
−64
[mm yr^{-1} K^{-1}]
c
δT
2.4
2.0
1.6
1.2
0.8
0.4
0.0
[mm yr^{-1} K^{-1}]
d
δE^{Penm}_{T}
e
δLW
2.4
1.2
0.0
−1.2
−2.4
[W m^{-2} K^{-1}]
f
δE^{Penm}_{LW}
32
16
0
−16
−32
[mm yr^{-1} K^{-1}]
g
δSW
h
δE^{Penm}_{SW}
i
δRH
0.8
0.4
0.0
−0.4
−0.8
[% K^{-1}]
j
δE^{Penm}_{RH}
k
δG
8
4
0
−4
−8
[W m^{-2} K^{-1}]
l
δE^{Penm}_{G}
m
δU
0.4
0.2
0.0
−0.2
−0.4
[m s^{-1} K^{-1}]
n
δE^{Penm}_{U}
60°N
30°N
0°
30°S
60°S
90°E
180°
90°W

Figure S3: a, Spatial pattern of projected evaporation changes per unit warming in CMIP models. **b,** Spatial pattern of estimated evaporation changes per unit warming using the Penman equation. **c,e,g,i,k,m,** Spatial pattern of projected changes in surface air temperature, net longwave flux, net shortwave flux, relative humidity, ocean heat uptake and windspeed. **d,f,h,j,l,n,** Spatial pattern of evaporation changes due to changes in individual factors estimated by the Penman equation.

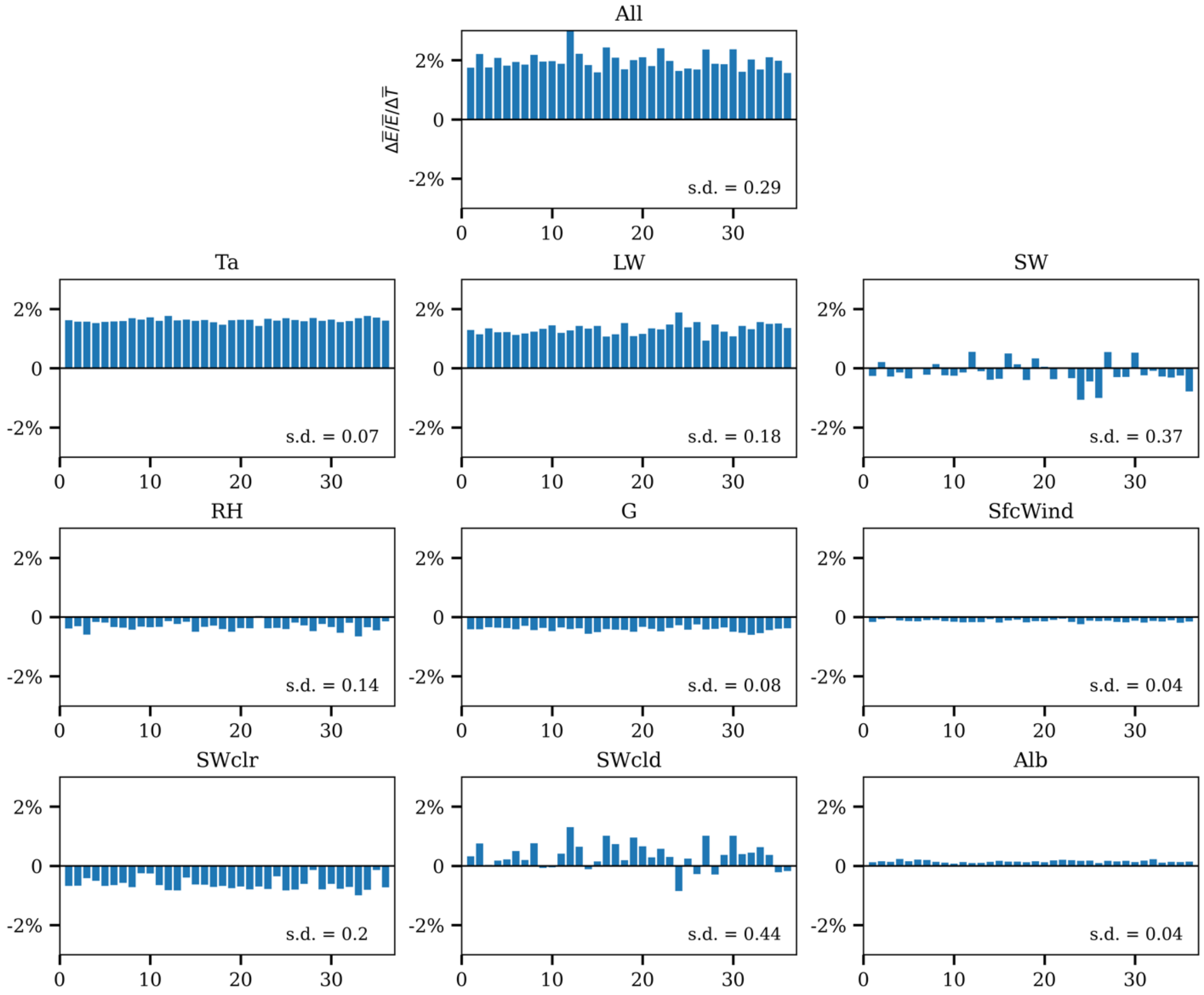

Figure S4: The changes in global mean ocean evaporation per unit warming in each climate mode due to changes in all and individual factors including temperature (Ta), longwave radiation (LW), shortwave radiation (SW), relative humidity (RH), ocean heat uptake (G), surface winds (SfcWind), clear-sky shortwave radiation (SWclr), cloud effect on shortwave radiation (SWcld) and albedo (Alb). The intermodel

s.d. is denoted. The x-axis indicates the model number.

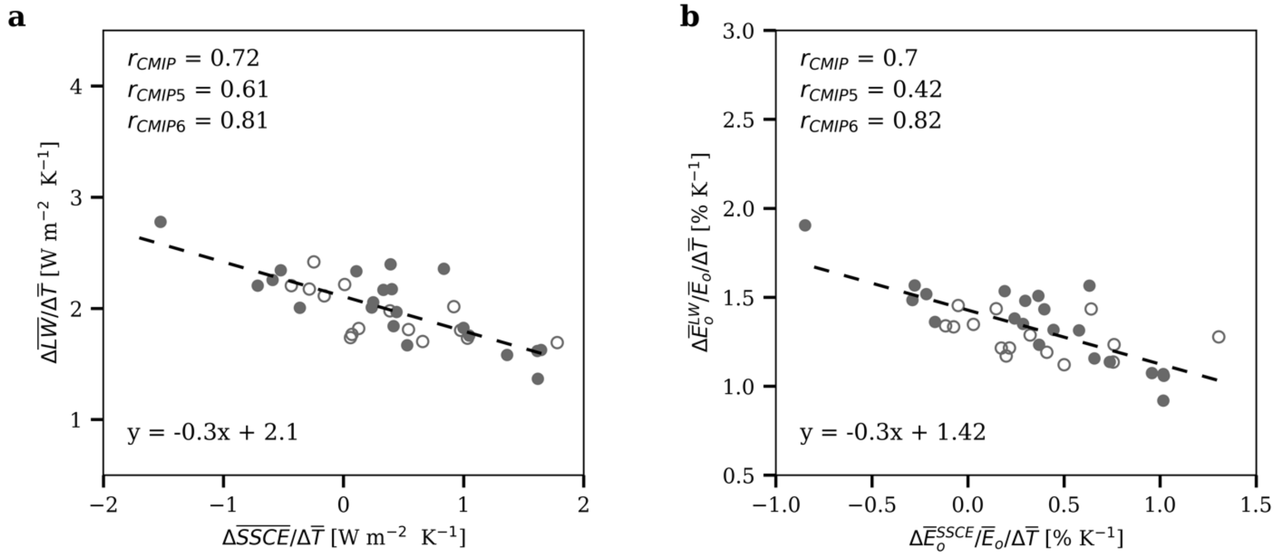

Figure S5: a, Intermodel scatterplot between $\Delta\overline{SSCE}/\Delta\overline{T}$ and $\Delta\overline{LW}/\Delta\overline{T}$. **b,** Intermodel scatterplot between the evaporation change per unit warming contributed by the SSCE change ($\Delta\overline{E}_o^{SSCE}/\overline{E}_o/\Delta\overline{T}$) and that contributed by the LW change ($\Delta\overline{E}_o^{LW}/\overline{E}_o/\Delta\overline{T}$). The open and filled dots are the CMIP5 and CMIP6 results, respectively.

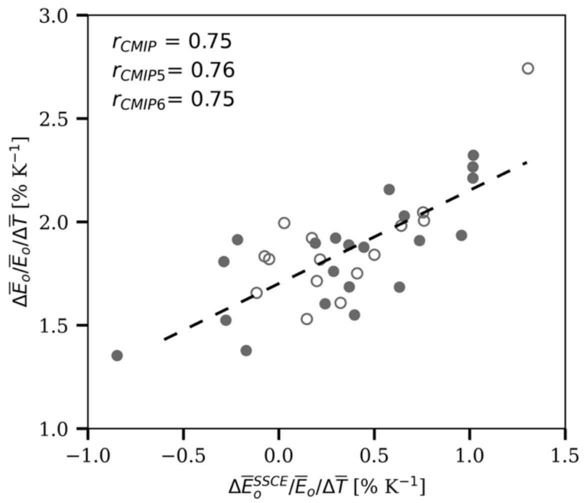

Figure S6: Intermodel scatterplot between the evaporation change contributed by the SSCE change estimated based on the Penman equation ($\Delta\bar{E}_o^{SSCE}/\bar{E}_o/\Delta\bar{T}$) and the model-projected evaporation change ($\Delta\bar{E}_o/\bar{E}_o/\Delta\bar{T}$). The open and filled dots are the CMIP5 and CMIP6 results, respectively.

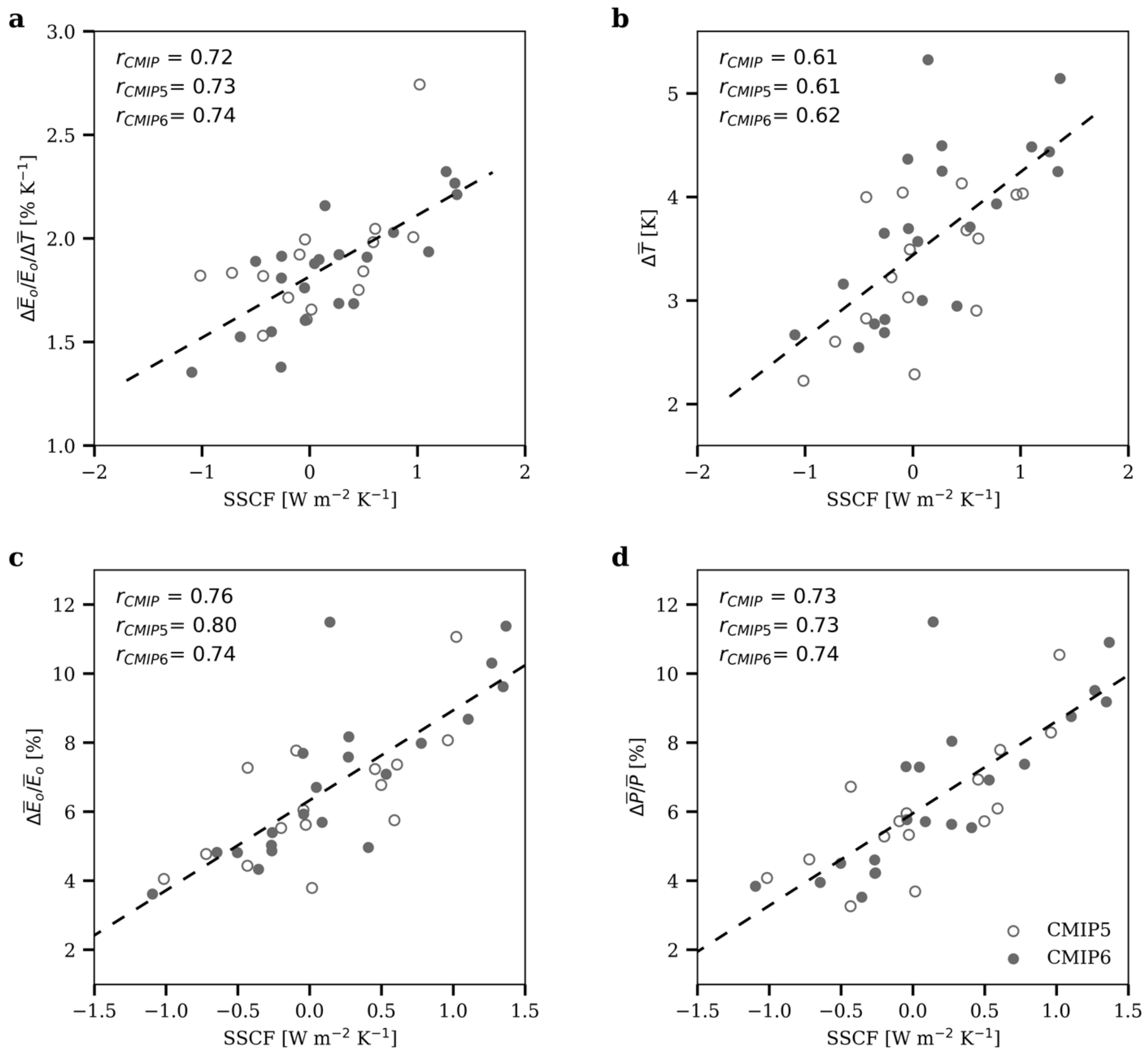

Figure S7: Same as Fig. 2a-d but with SSCF diagnosed from Abrupt4xCO2 as the x axis.

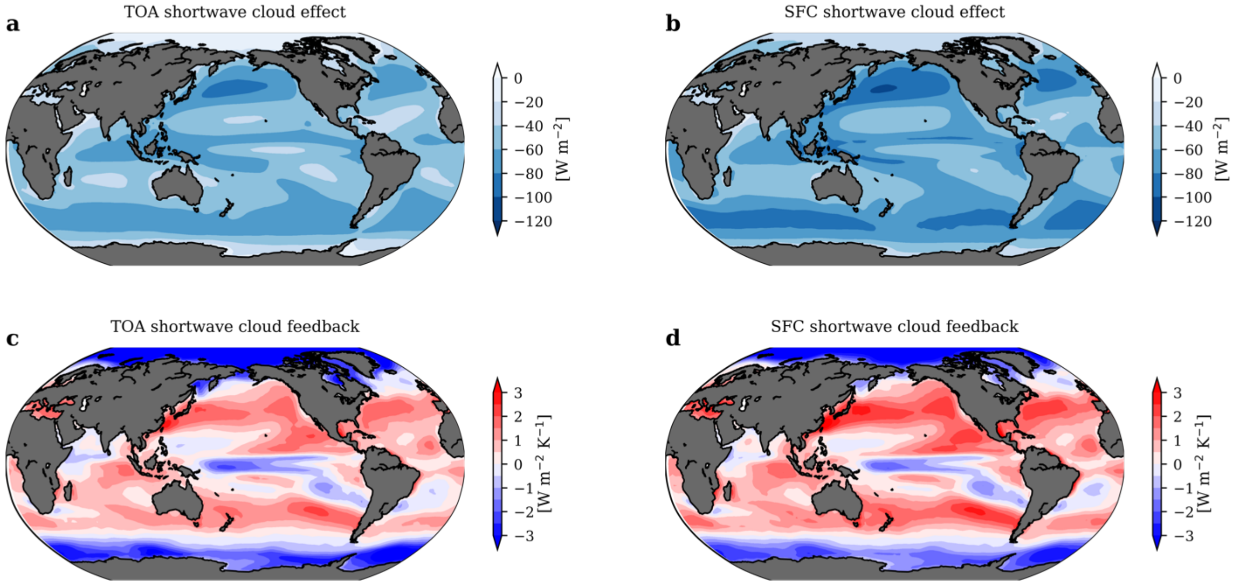

Figure S8: The shortwave cloud effect and feedback at the TOA and SFC.

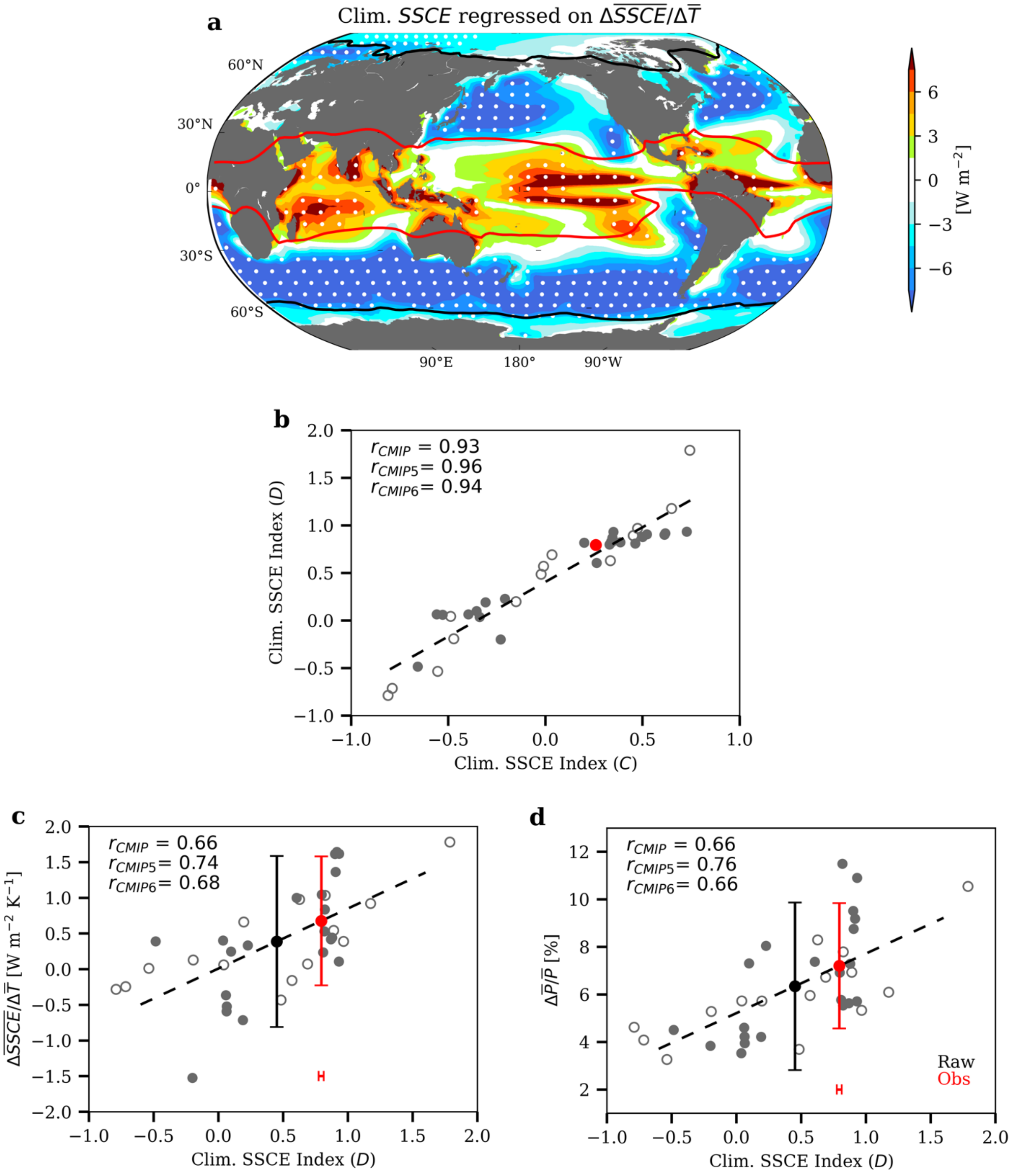

Figure S9: Same as Fig. 4 but using the simple climatological index of SSCE, Index D. The red contour in **a** indicates the contour of 25°C.

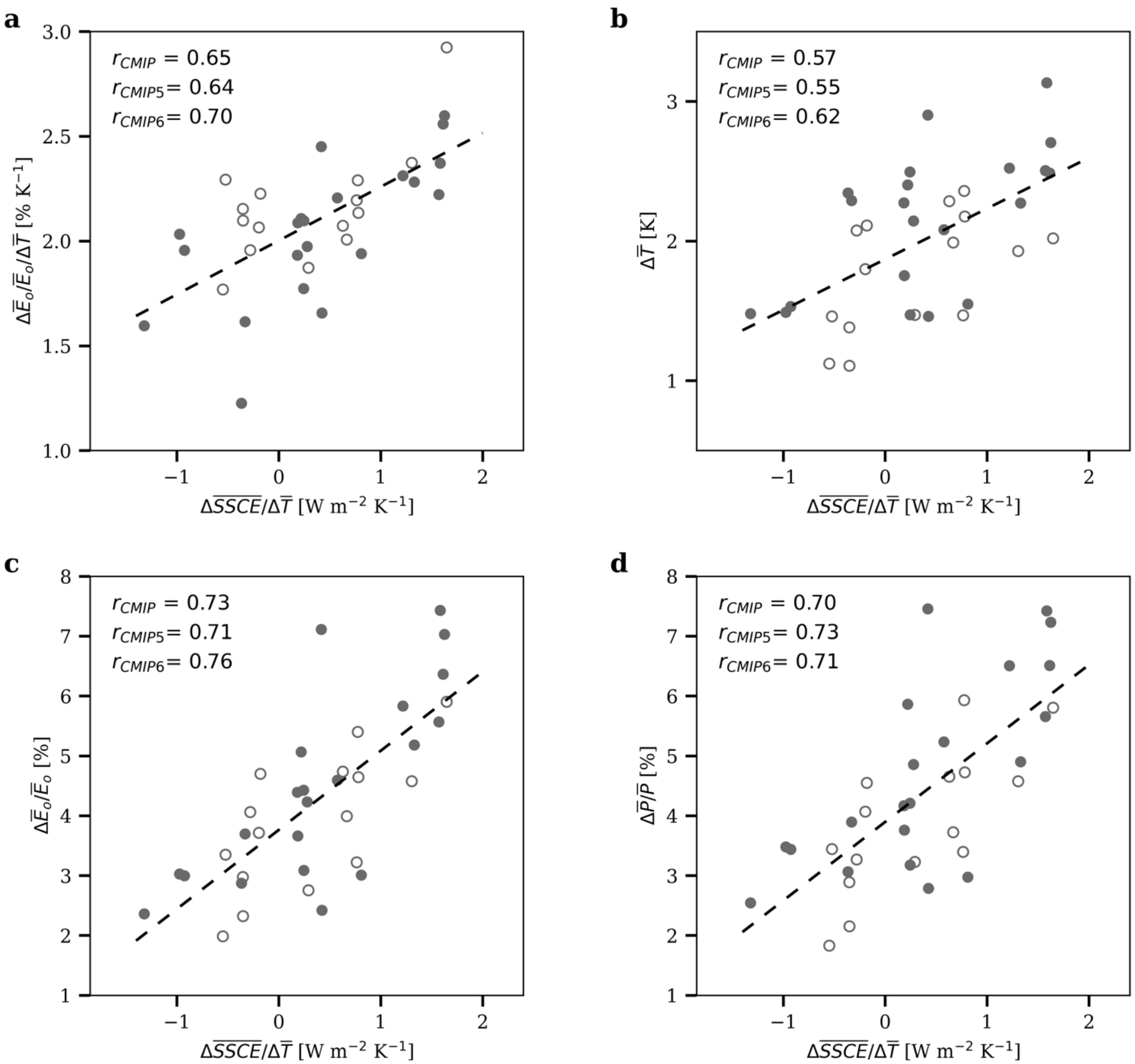

Figure S10: Same as Fig. 2a-d but for the RCP4.5/SSP2-4.5 emission scenario.

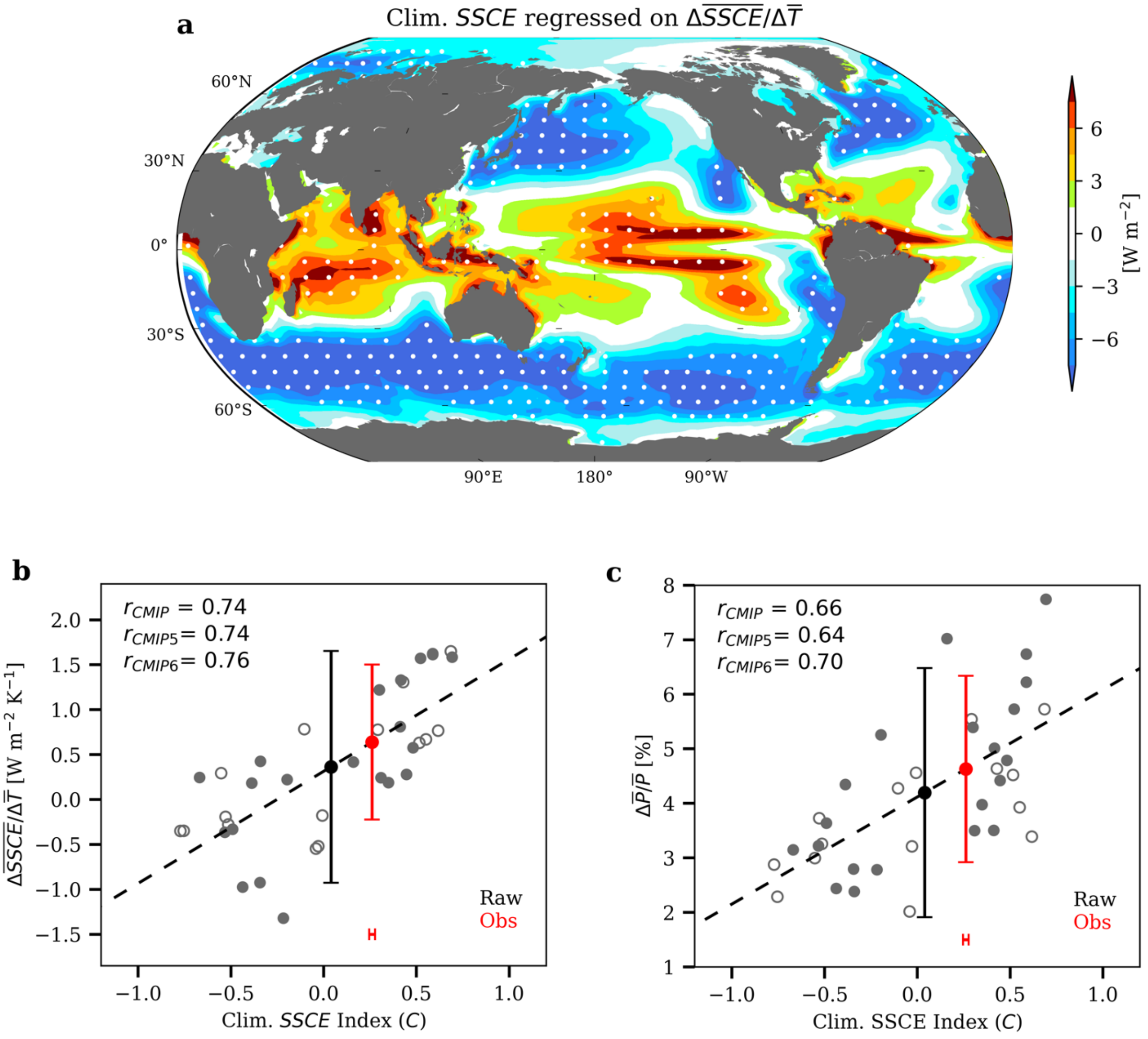

Figure S11: Same as Fig. 4 but for the RCP4.5/SSP2-4.5 emission scenario.

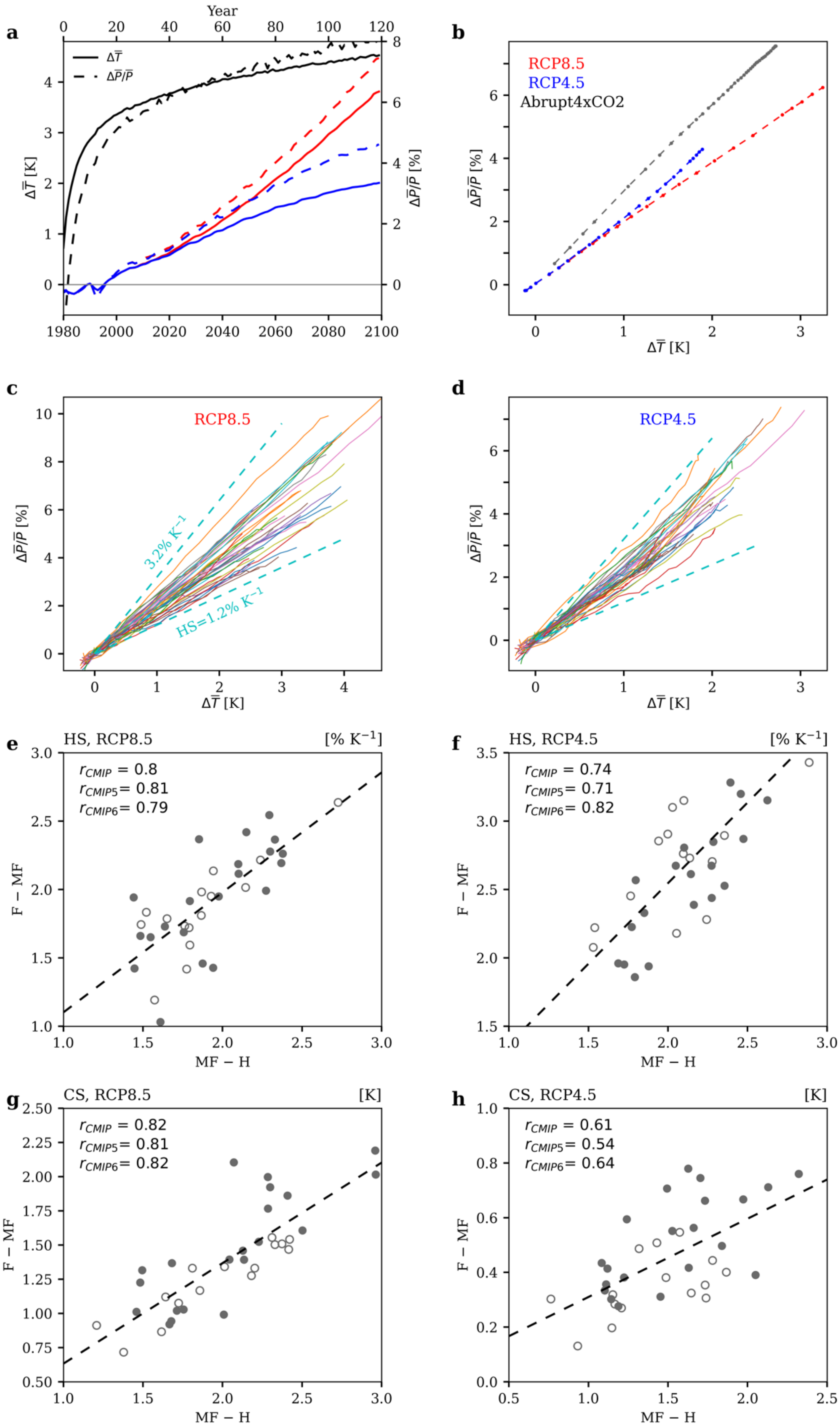
a
b
c
d
e
f
g
h
Year
$\Delta\overline{T}$
$\Delta\overline{P}/\overline{P}$
$\Delta\overline{T}$ [K]
$\Delta\overline{P}/\overline{P}$ [%]
RCP8.5
RCP4.5
Abrupt4xCO2
3.2% K^{-1}
HS=1.2% K^{-1}
HS, RCP8.5
[% K^{-1}]
r_{CMIP} = 0.8
r_{CMIP5}= 0.81
r_{CMIP6}= 0.79
HS, RCP4.5
[% K^{-1}]
r_{CMIP} = 0.74
r_{CMIP5}= 0.71
r_{CMIP6}= 0.82
CS, RCP8.5
[K]
r_{CMIP} = 0.82
r_{CMIP5}= 0.81
r_{CMIP6}= 0.82
CS, RCP4.5
[K]
r_{CMIP} = 0.61
r_{CMIP5}= 0.54
r_{CMIP6}= 0.64
F − MF
MF − H

Figure S12: a, Temporal evolution of the global mean temperature ($\Delta\overline{T}$, solid) and precipitation ($\Delta\overline{P}/\overline{P}$, dashed) under RCP8.5/SSP5-8.5 (red), RCP4.5/SSP2-4.5 (blue) and Abrupt4xCO2 (black). **b,** Relationship between changes in temperature and precipitation (the slope indicates hydrological sensitivity $\Delta\overline{P}/\overline{P}/\Delta\overline{T}$) under RCP8.5/SSP5-8.5 (red), RCP4.5/SSP2-4.5 (blue) and Abrupt4xCO2 (black). **c,** Relationship between changes in temperature and precipitation under RCP8.5/SSP5-8.5 for individual models. The slopes of HS=1.2% K-1 and HS=3.2% K-1 are plotted for reference. **d.** As c but for RCP4.5/SSP2-4.5. **e.** Intermodel scatterplot of HS in the early (Mid-Future minus Historical, i.e., 2049-2069 minus 1979-2005, denoted as MF - H) and late (Future minus Mid-Future, i.e., 2080-2100 minus 2049-2069, denoted as F - MF) periods of RCP8.5/SSP5-8.5. **f.** As e but for RCP4.5/SSP2-4.5. **g,h,** As e,f but for CS. The open and filled dots are the CMIP5 and CMIP6 results, respectively.

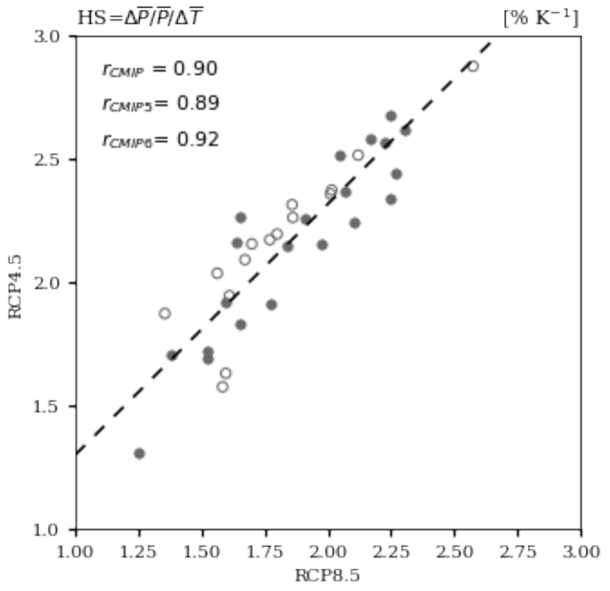

Figure. S13: Intermodel scatterplot between HS's in RCP8.5/SSP5-8.5 and RCP4.5/SSP2-4.5.

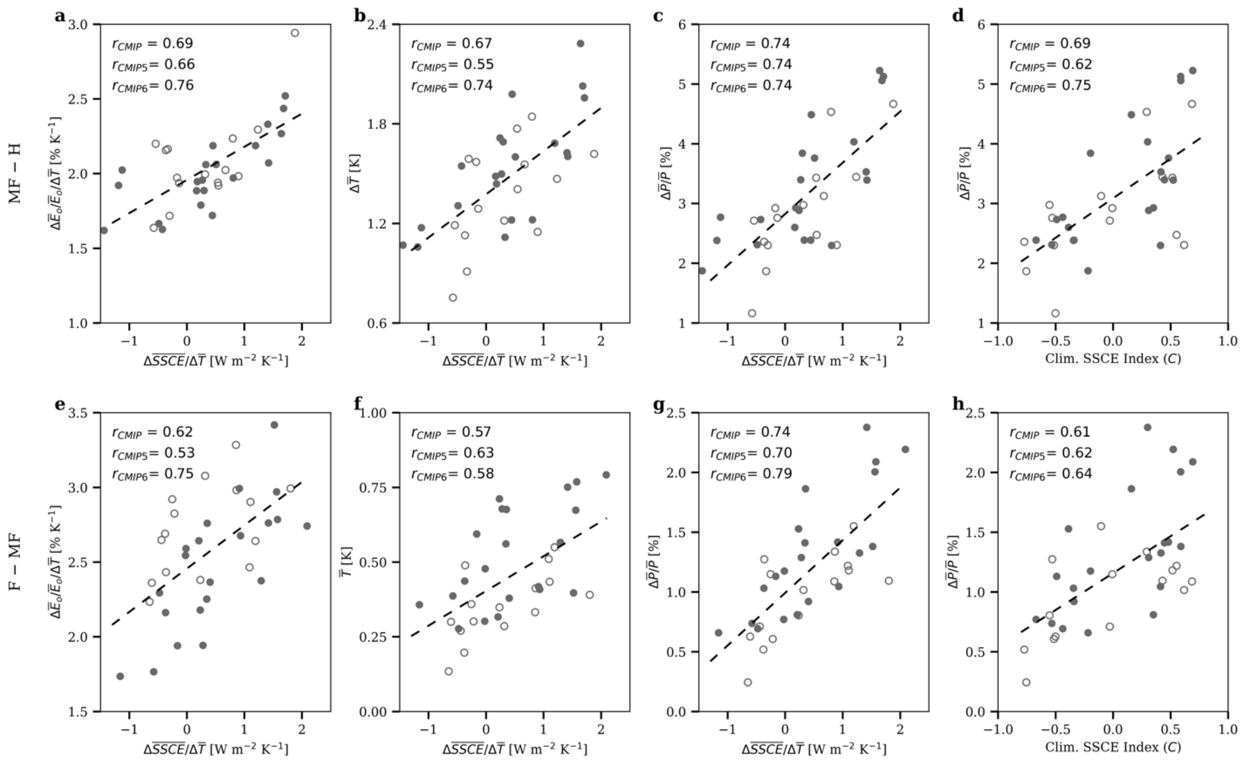

Figure S14: **a,e** Intermodel scatterplot between $\Delta\overline{SSCE}/\Delta\overline{T}$ and $\Delta\bar{E}_o/\bar{E}_o/\Delta\bar{T}$. **b,f** Intermodel scatterplot between $\Delta\overline{SSCE}/\Delta\overline{T}$ and $\Delta\bar{T}$. **c,g** Intermodel scatterplot between $\Delta\overline{SSCE}/\Delta\overline{T}$ and $\Delta\bar{P}/\bar{P}$. **d,h** Intermodel scatterplot between Index C and $\Delta\bar{P}/\bar{P}$. **a-d** are for the early period of the 21st century under RCP4.5/SSP2-4.5 (Mid-Future minus Historical, 2049-2069 minus 1979-2005) while **e-h** are for the late period of the 21st century under RCP4.5/SSP2-4.5 (Future minus Mid-Future, 2080-2100 minus 2049-2069). The open and filled dots are the CMIP5 and CMIP6 results, respectively.

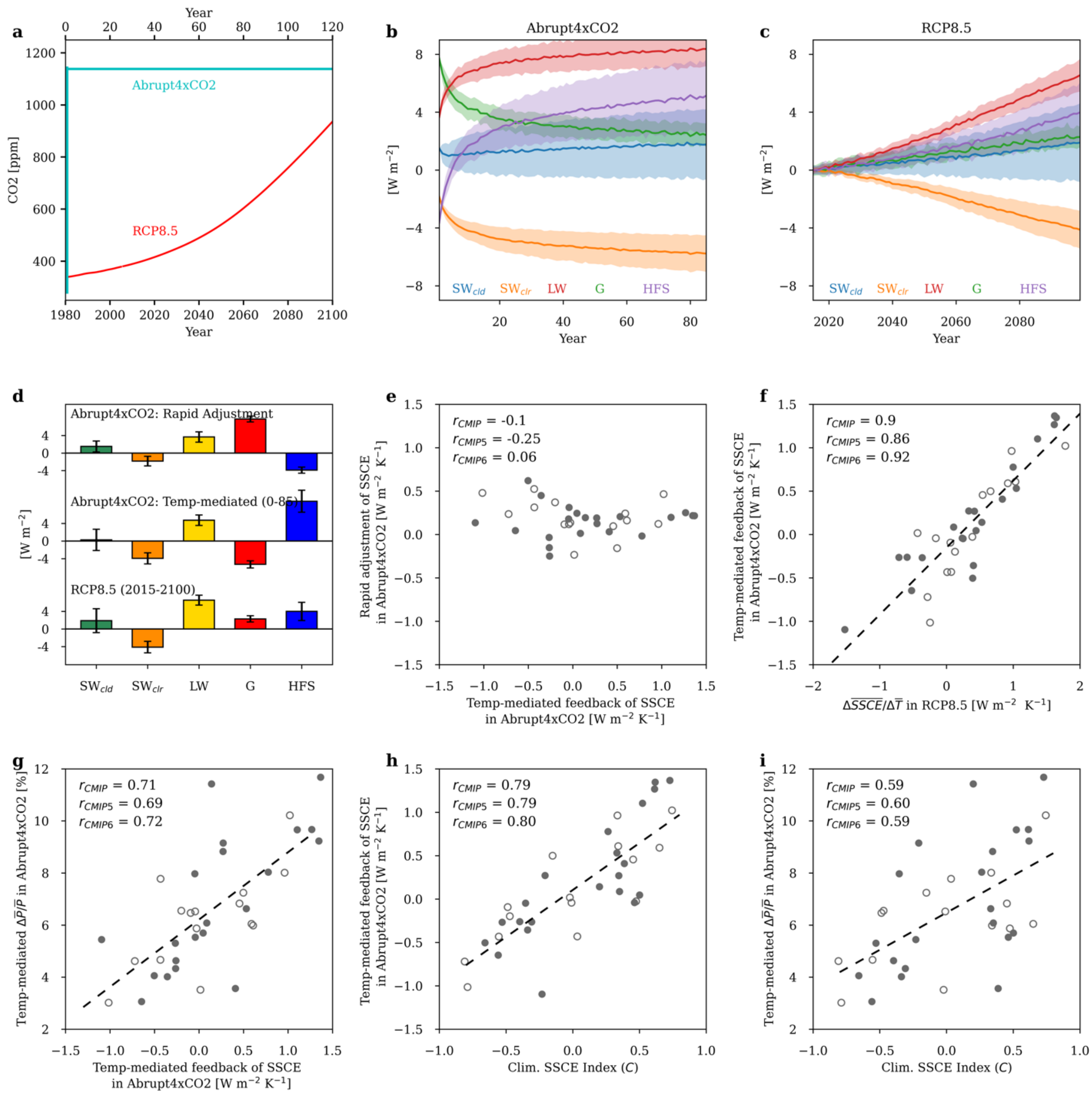

Figure S15: a, Temporal evolutions of the atmospheric CO2 concentration under Abrupt4xCO2 and RCP8.5/SSP5-8.5. **b,** Temporal evolutions of different terms associated with surface energy balance, i.e., global mean SW_{cld}, SW_{clr}, LW, G and HFS, to Abrupt4xCO2. The solid line shows the ensemble model mean and the shading indicates the intermodel s.d. **c,** As b but for RCP8.5/SSP5-8.5. **d,** Ensemble model mean and intermodel s.d. of the changes in SW_{cld}, SW_{clr}, LW, G and HFS under Abrupt4xCO2 (separated into rapid adjustment and temperature-mediated effects) and under RCP8.5/SSP5-8.5. **e,** Intermodel scatterplot between rapid adjustment and temperature-mediated feedback of SSCE under Abrupt4xCO2.

To compare their relative effects, the effect of rapid adjustment has been normalized by global ocean warming. **f,** Intermodel scatterplot between the temperature-mediated feedback of SSCE diagnosed from Abrupt4xCO2 (i.e., SSCF) and $\Delta\overline{SSCE}/\Delta\overline{T}$ under RCP8.5/SSP5-8.5. **g,** Intermodel scatterplot between SSCF and the temperature-mediated precipitation increase under Abrupt4xCO2. **h.** Intermodel scatterplot between Index C and SSCF. **i,** Intermodel scatterplot between Index C and the temperature-mediated precipitation increase under Abrupt4xCO2. The open and filled dots are the CMIP5 and CMIP6 results, respectively.
